# Post-market quality monitoring of medicines in Christian Health Association of Ghana health institutions

**DOI:** 10.4314/gmj.v59i3.3

**Published:** 2025-09

**Authors:** Daniel K Arhinful, Peter Yeboah, James Duah, Maxwell A Antwi, Alex Attachey, Eric Karikari-Boateng, Alhassan M Awal, Tobias F Rinke de Wit, Irene A Kretchy

**Affiliations:** 1 Department of Epidemiology, Noguchi Memorial Institute for Medical Research, University of Ghana, Legon; 2 Christian Health Association of Ghana, No. 21 Jubilee Well Street, Labone, P.O. Box AN7316, Accra-North; 3 Pharmaccess Foundation, 5 Dakar Avenue, GA-416-5058. East Legon, Accra, Ghana; 4 Centre for Laboratory Services and Research, Drug Lab Dept. Food & Drugs Authority (FDA), Accra, Ghana; 5 Amsterdam Institute for Global Health and Development, Dept. Global Health, University of Amsterdam, The Netherlands; 6 Department of Pharmacy Practice and Clinical Pharmacy, School of Pharmacy, University of Ghana, Legon

**Keywords:** medicines quality assurance, Truscan, post-market surveillance, supply chain, Minilab

## Abstract

**Objective:**

To determine the quality of selected frequently used medicines in CHAG facilities in a LMIC country setting.

**Design:**

Quality testing of collected samples of generic medicines in health facility pharmacies.

**Setting and participating facilities:**

The study evaluated the quality of 639 representative samples of 14 generic products using a TruScan Raman (RM) analyser and Minilab in 62 CHAG facilities across five administrative regions of Ghana.

**Results:**

Out of 639 samples of various branded generics of the 14 product samples tested in the field using the Truscan RM analyser, 210 products (32.8%) failed the test. All samples from ceftriaxone injection 1 g, ciprofloxacin 500 mg, metronidazole 200 mg, and metformin 500 mg passed the Truscan RM test. High passes were also recorded for paracetamol 500 mg (96.6%), artemether/lumefantrine 80/480 mg (95.8%), and oral rehydration salts (94%). Conversely, all the forty-two (42) samples obtained and tested for flucloxacillin 250 mg failed the Truscan analyser test. Relatively high failures were also recorded for lisinopril 10 mg (90.5%) and albendazole 400 mg (89.8%). All samples submitted for secondary screening using Minilab analysis showed the presence of their respective active pharmaceutical ingredients as indicated on their respective labels.

**Conclusion:**

The Truscan/Minilab combination is reasonably affordable and efficient for undertaking post-market monitoring of the quality of essential medicines in Ghanaian health facilities. For future application of Truscan, a check of standard spectra is essential, and the choice of tracer medicines should include those with limited fluorescence materials in formulations and a relatively high percentage of active ingredients.

**Funding:**

This study was funded by Pharmaccess Foundation Ghana and the Netherlands

## Introduction

The World Health Organisation (WHO) has defined substandard medical products as “authorised medical products that fail to meet either their quality standards or their specifications, or both”,^1^ while *falsified* medical products are “products that deliberately/fraudulently misrepresent their identity, composition or source.”.^2^ The use of falsified/substandard medicines is a global concern, especially in LMICs where pharmaceutical supply chain processes may be less robust.^3^,^4^ Falsified and substandard drugs are a serious problem in Africa as in many other parts of the world, leading to unnecessary deaths and severe public health and economic damage. Various studies^5^,^6^,^7^ have revealed that middlemen, illegal trade, falsified and low-quality products depict the medicines supply chain for healthcare providers in Africa. The falsified/substandard trade is worth about $1 billion a year, and nearly 700,000 people are estimated to die from falsified and substandard malaria and tuberculosis medicines each year globally.^8^ According to a report by the Organisation for Economic Cooperation and Development (OECD), the global trade in counterfeit and pirated goods, including pharmaceuticals, was worth nearly USD 500 billion in 2019. The report estimates that counterfeit pharmaceuticals make up a significant portion of this trade. The presence of substandard medicines can be attributed to manufacturing errors, inadequate storage, or poor distribution practices. The production of falsified medicines is often driven by economic exploitation.^3^,^9^

A 2013 WHO report indicated that in Africa, 100,000 deaths are due to trading in counterfeit drugs.^10^ The WHO Global Surveillance and Monitoring System reported that 42% of the 1,500 cases of substandard and falsified medical products recorded between 2013 and 2017 were from the Africa region. Antibiotics and antimalarials were the most frequently reported pharmaceutical products, accounting for approximately 36% of all reported products. The study also estimated 10.5% failure rates of substandard and falsified medical products in LMICs) In 2017, another WHO report estimated that 1 in 10 medical products in low- and middle-income countries (LMICs), including Ghana, are substandard or falsified, with antibiotics and antimalarials being particularly susceptible.^4^ Following the damning report, a number of the affected countries initiated measures to rectify the situation. In Ghana, the government, with support from the US Pharmacopoeia and WHO, launched a thorough drug sampling and testing programme that regularly published its findings. Regulatory penalties, such as fines and recalls, were imposed on non-compliant importers or manufacturers. In fact, the reinforced and sustainable post-market surveillance programme helped the Government improve the systems for preventing, detecting, and responding to the situation of fake/substandard drugs on the Ghanaian market.^12^ Ingesting falsified/substandard medicines can potentially cause more harm to patients than if they (the patients) had not sought any medical treatment at all. Substandard and falsified medical products may fail to treat the diseases for which they were intended, and they can also contribute to antimicrobial resistance and drug-resistant infections, leading to a loss of confidence in medicines, healthcare providers, and health systems.^13^,^14^

In Ghana, antimalarials are among the most faked medicines on the market. For example, 13 out of 14 (93%) Artemisinin-based medicines obtained from pharmacies and licensed chemical sellers in Kumasi, Ghana's second largest city, had either too low or too high active pharmaceutical ingredient (API) concentrations, which is the active ingredient contained in a medicine.^15^ Also in 2012, the fourth round of the United States Pharmacopoeia quality monitoring (USP/FDA PQM) Anti-Malaria project executed by the Ghana FDA observed a failure rate of 6.3% for antimalarial preparations involving 370 samples of antimalarial preparations on the market screened with Minilabs, of which 165 were subjected to full monograph analysis.^16^ Another study found that about 9% of antimalarials for public consumption in Ghana had gone beyond their expiry date and 35% were substandard.^17^ The critical public health implications of such findings are that, while too low levels of the active ingredients may lead to therapeutic ineffectiveness and artemisinin resistance, too high levels of active ingredients, expired and substandard products may lead to drug toxicities due to high levels of degradants.

The Food and Drugs Authority in Ghana is mandated by law to ensure the quality, safety, and efficacy of all pharmaceutical products marketed in Ghana, as well as packaged food, cosmetics, chemical devices, and household chemicals. Once the Authority has granted a product marketing authorisation, the quality of subsequent batches of the product, whether locally manufactured or imported, is to be regularly assessed through post-market surveillance activities and intelligence.

However, due to financial constraints and the overstretched capacity of the Food and Drug Authority's quality control laboratory services, only a few categories of medicines are selected for testing at a time. This has implications for the widespread assurance of the quality of medicines, as some manufacturers subsequently reduce the active ingredients of medicines after batches that were initially submitted to the FDA and had passed the required standards. Healthcare facilities in Ghana have limited reliable measures in place to check the quality of medicines currently in use. A previous study in Ghana described three options (GPHF Minilab, Colourimetry, and Counterfeit Drug Indicator) but recommends alternatives and additional research.^19^ The current study concentrated on the potential of a novel methodology, Truscan, for use in peripheral pharmacies as an alternative to centralised laboratory screening. New technologies such as TruScan and mPedigree's network are now being used actively to identify and fight the substandard drug trade. Such technological products have empowered healthcare providers and consumers elsewhere to protect themselves by sending simple text messages to confirm the genuineness of drugs.^20^

This study assessed the quality levels of selected frequently used medicines in some private not-for-profit healthcare facilities in a low- and middle-income country setting in Ghana using a TruScan Raman (RM) analyser and Minilab.

## Methods

A multistage sampling procedure was applied to select ecological and geographic areas comprising five administrative regions from the pre-2019 ten regions of Ghana. We initially planned to study 60 facilities, but due to operational reasons, 62 facilities were surveyed in CHAG member health facilities nationwide. The study then randomly selected and digitally checked the quality of 639 representative samples of 14 generic products (Supplementary table) from various dosage forms and strengths, as specified in the Essential Medicines List (EML) of Ghana, in 62 CHAG facilities participating in or earmarked for participation in the Med4All programme.^1^ After visual inspection, the sampled products were tested on-site using a handheld portable spectroscopic TruScan™ Raman (RM) analyser. The following information was recorded: type of product (name, active ingredients), sampling level, sampling date, sample sequential number, geographic zone, Region and facility type from which products were sampled. For the TruScan assessment, each product was placed in the sample compartment from which a spectrum is generated by emitting a laser beam from the device, which is placed in contact with the sample compartment. The spectrum generated for each sample was compared to a reference library of spectra.

Data collected with the TruScan™ RM analyser were stored on a server and were only available and accessible to key members of the study team for analysis and reporting. The study's results were aggregated, and information about individual participating facilities was kept confidential for ethical reasons. The TruScan™ RM analyser is equipped with an enhanced data management function. The spectrum generated was automatically analysed using a chemometric technique (Principal Component Analysis) to identify and compare with the reference spectrum. The instrument then uses multivariate residual analysis as a decision engine to determine results that match the reference spectrum (pass) or deviation from the reference spectrum (fail). Following the data acquisition using the TruScan™ RM analyser in this study, the results were then keyed into an Excel spreadsheet. A simple descriptive analysis was conducted by calculating the percentage of passed and failed results for the fourteen generic products surveyed in the study.

Some products that failed the TruScan test were sampled, transported, and submitted for further screening by the Ghana Food and Drugs Authority (FDA) to determine the presence or absence of the active pharmaceutical ingredients (APIs) using the GPHF MiniLab analysis, as per the manufacturer's instructions. Approval for the study was obtained from the Research Institutional Review Board of the Christian Health Association of Ghana (No: CHAGIRB 05022020 Approved on 15^th^ September 2020). Local managers of the chosen CHAG health facilities were also contacted for permission before visiting the facilities to obtain the products. Informed consent was also obtained after the facility's contact persons had gone through the information form and signed the consent form, which was available in English.

## Results

### Summary results using the Truscan RM analyser

Of the fourteen generic products, paracetamol 500 mg was the most commonly available (59 out of 62 possible) in the facilities surveyed, followed by oral rehydration salts (55 out of 62 possible). Artemether lumefantrine 80/480MG was the least available (24 out of possible 62) followed by ferrous sulphate 200MG (37 out of possible 62).

Out of the total of 639 samples of various brands of the 14 generic product samples tested in the field using the Truscan RM analyser, 210 products (32.8%) failed the Truscan test (Table 1). All samples obtained for 4 generic products, namely ceftriaxone injection 1g (50), ciprofloxacin 500mg (50), metronidazole 200mg (48) and metformin 500mg (45), passed the Truscan RM test. High passes were also recorded for paracetamol 500mg (96.6%), artemether-lumefantrine 80/480mg (95.8%) and oral rehydration salt (94% %).

**Table 1:** Minilab test results for samples which failed the TruScan

No	Sample ID	Generic Name	Colour	Size	Intensity	shape	Travel distance	Rf value	Rf value for reference std spot	Conclusion
**1**	FDA/LSD21/DG0820	Paracetamol tablets	+	+	+	+	+	0.74	0.74	Passed
**2**	FDA/LSD21/DG0821	Amoxicillin and clavulanic acid tablets	+	+	+	+	+	0.36^a^	0.36^a^	Passed
								0.08^c^	0.08^c^	
**3**	FDA/LSD21/DG0822	Lisinopril tablets	+	+	+	+	+	0.49	0.49	Passed
**4**	FDA/LSD21/DG0823	Albendazole tablets	+	+	+	+	+	0.72	0.72	Passed
**5**	FDA/LSD21/DG0824	Diclofenac injection	+	+	+	+	+	0.30	0.30	Passed
**6**	FDA/LSD21/DG0825	Albendazole tablet	+	+	+	+	+	0.73	0.72	Passed
**7**	FDA/LSD21/DG0826	Lisinopril tablet	+	+	+	+	+	0.49	0.49	Passed
**8**	FDA/LSD21/DG0827	Amoxicillin and clavulanic acid tablets	+	+	+	+	+	0.36^a^	0.36^a^	Passed
								0.08^c^	0.08^c^	
**9**	FDA/LSD21/DG0828	Albendazole tablet	+	+	+	+	+	0.63	0.63	Passed

a amoxicillin spot

c clavulanic acid spot

+ The product spot in the chromatogram obtained with the test solution corresponded in terms of colour, size, intensity, shape and travel distance to that in the chromatogram obtained with the lower and higher standard solution.

The Rf value representing relative movement of sample spot to the solvent front which is unique to the analyte were the same as the Rf value for the reference standard spot.

**Figure 1:**
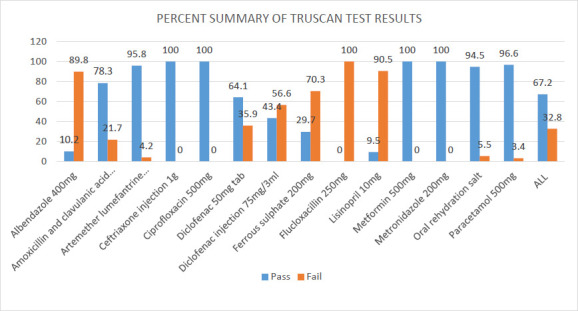
Summary of Truscan test results of various brands of fourteen generic products tested

Conversely, all 42 samples obtained and tested for flucloxacillin 250mg failed the Truscan analyser test. Relatively high failure rates were also recorded for lisinopril 10 mg (90.5%), albendazole 400 mg (89.8%), and ferrous sulphate 200 mg (70.3%). Figure 1 provides the summary, which is further detailed in Supplementary Table 1.

### Minilab validation by the FDA

To further analyse the TruScan-failed samples, nine representative samples were selected and submitted for minilab analysis at the Centre for Laboratory Services and Research at the FDA. The criteria for selecting samples for further analysis at the FDA were partly purposive, aiming to select all samples with higher failure rates. However, flucloxacillin could not be included in the confirmatory tests at the FDA because powdered samples of the product from two different facilities from two different manufacturers were all caked and therefore compromised for further testing and analysis. The minilab validation analysis showed that all (100%) of the samples contained the respective APIs stated on their respective labels. Table 1 below provides the overview.

## Discussion

This study deployed the TruScan RM analyser as an efficient post-market surveillance tool to rapidly determine the quality of selected frequently used medicines in CHAG facilities in a LMIC setting where standard spectra were available in its library for comparison. Although specific sensitivity and specificity values were not readily available, the analyser's advanced chemometric algorithms ensured accurate material identification in sealed packaging. Also, even though the analyser did not yield specific quantitative values, its deployment yielded pass/fail results within seconds, with an option for STRONG PASS/WEAK PASS and STRONG FAIL/WEAK FAIL results in a fast and straightforward duration using minimal samples. Two out of three of the drug samples collected at the CHAG facilities in Ghana (429/639) passed the Truscan test, indicating that those samples contained active pharmaceutical ingredients (API's) as stated in the manufacturer's specifications.

A third of the samples obtained, however, failed the Truscan test. All flucloxacillin 250 mg generic samples failed the test, while lisinopril 10 mg, albendazole 400 mg, and ferrous sulphate 200 mg generics also recorded high failures. The failure outcome of the samples using the TruScan test could be due to sub-standardisation or falsification if the samples do not align with the expected chemical composition of the standards, or if the samples contain adulterants, impurities, or contaminants.^21^

Yet when the failed samples were analysed with the GPHF-MiniLab, all the samples passed based on the sample spot in the chromatogram obtained with the test solution, which corresponded in terms of colour, size, intensity, shape, and travel distance with that of the reference solution. These results are similar to those of some studies, where products with low doses of active pharmaceutical ingredients and fixed-dose combinations of active ingredients have been reported to yield false negatives.^22^,^23^ The precision of the handheld Truscan could thus depend on the nature and the strength of the API in the finished pharmaceutical product tested, which could affect the spectrum generated, as in the case of Lisinopril.^24^

Another possible factor that led to Truscan failures might have been poor storage. For example, two different samples of flucloxacillin 250 mg manufactured locally by two other companies and collected from different facilities were caked when removed from the capsule shell. Indeed, the two products were both caked at the time of the confirmatory tests and therefore compromised for further testing and analysis. The caking could be due to the highly hygroscopic nature of the powdered formulation of the drug reacting with moisture during its storage before or after leaving the manufacturer. This probably affected the integrity of the product and further justified the failed result.^25^

Furthermore, some of the products failed the Truscan test due to batch problems; the batches obtained in the field differed from those programmed in the Truscan device received from the FDA. The study took necessary steps to clarify with the Drug Evaluation and Registration Department of the FDA to ensure that the drug batches analysed in the lab were the batches submitted to the Drug Evaluation and Registration Department. It turned out that not always were the batch numbers from field samples the same as those of the controls.

To validate the Truscan failure results, the selected products submitted for MiniLab analysis at the FDA were found to contain the respective APIs, as indicated in the MiniLab manual. However, since both TruScan and Minilab are qualitative instruments, it was not possible to ascertain the actual quantities of the API. Confirmation of quantitatively correct active ingredients will require a more robust confirmatory analysis, such as high-performance liquid chromatography (HPLC) analysis, to determine the actual API quantities.

### Study limitations

Although both the Truscan and minilab are reasonably affordable and efficient for undertaking post-market quality monitoring of medicines to assure the quality of essential medicines in health facilities, the Truscan is only an efficient post-market surveillance tool, provided standard spectra are available in its library for comparison. Additionally, as observed elsewhere, the precision of the handheld Truscan depends on the nature and strength of the API in the finished pharmaceutical product being tested.^26^ When scanning for a low-dose product where the active ingredient was small or low, the chances are that you usually would not get a good spectrum, as was seen in the lisinopril 10 mg and nifedipine products.

Based on the study findings, the following recommendations are made.

- From a regulatory perspective, TruScan could be used as an efficient post-market surveillance tool, provided standard spectra are available in its library for comparison, and low auto-fluorescence tracer drugs are selected with relatively higher content of active ingredients. This means that in the Ghanaian context, where essential medicines for common conditions have multiple generic formulations, unless each of these generic formulations has been scanned and recorded in the TruScan library, authentication will not always be possible.
- In a similar future post-market surveillance to use the TruScan test as a routine screening tool, the test results should be compared with other analytical methods like the GPHF-MiniLab, and further using confirmatory methods such as the High-Performance Liquid Chromatography (HPLC) to ascertain the true positives, false positives, true negatives, and false negatives.
- The regulatory recommendation from the study is that ‘the best should not be the enemy of the good’. In the Ghanaian context, when using Truscan for point-of-care post-market surveillance of medicines, it will be more efficient to select a set of tracer drugs that (1) do not have multiple generics and/or (2) have high dose drugs. Under those circumstances, drug batch numbers will play a minimal role, and using the Minilab to evaluate drugs that fail Truscan will be sufficient.

## Conclusion

The combination Truscan/GPHF-Minilab is reasonably affordable and efficient for undertaking post-market quality monitoring of medicines in Ghana.

## Footnotes

1 Altogether, a total of 14 different medicines were expected to be tested across the sixty facilities to make a total of 840 but due to lack of availability for some of the targeted medicines in some of the facilities, a total 639 dosage units of the targeted samples were obtained, sampled and tested across all 62 facilities.
